# Construction and Analysis of Siberian Tiger Bacterial Artificial Chromosome Library with Approximately 6.5-Fold Genome Equivalent Coverage

**DOI:** 10.3390/ijms15034189

**Published:** 2014-03-07

**Authors:** Changqing Liu, Chunyu Bai, Yu Guo, Dan Liu, Taofeng Lu, Xiangchen Li, Jianzhang Ma, Yuehui Ma, Weijun Guan

**Affiliations:** 1 Institute of Animal Science, Chinese Academy of Agricultural Sciences, Beijing 100193, China; E-Mails: lcq7813@hotmail.com (C.L.); baichunyu001@hotmail.com (C.B.); taofenglu@gmail.com (T.L.); nobelli@gmail.com (X.L.); Yuehui_Ma@hotmail.com (Y.M.); 2 Department of Bioscience, Bengbu Medical College, Bengbu 233000, China; E-Mail: ily0720@hotmail.com; 3 The Siberian Tiger Park of Heilongjiang, Harbin 150028, China; E-Mail: liudan1964@gmail.com; 4 College of Wildlife Resource, Northeast Forestry University, Harbin 150028, China; E-Mail: jianzhangma01@gmail.com

**Keywords:** Siberian tiger, *Panthera tigris altaica*, BAC library, genomic research, microsatellite marker

## Abstract

Bacterial artificial chromosome (BAC) libraries are extremely valuable for the genome-wide genetic dissection of complex organisms. The Siberian tiger, one of the most well-known wild primitive carnivores in China, is an endangered animal. In order to promote research on its genome, a high-redundancy BAC library of the Siberian tiger was constructed and characterized. The library is divided into two sub-libraries prepared from blood cells and two sub-libraries prepared from fibroblasts. This BAC library contains 153,600 individually archived clones; for PCR-based screening of the library, BACs were placed into 40 superpools of 10 × 384-deep well microplates. The average insert size of BAC clones was estimated to be 116.5 kb, representing approximately 6.46 genome equivalents of the haploid genome and affording a 98.86% statistical probability of obtaining at least one clone containing a unique DNA sequence. Screening the library with 19 microsatellite markers and a SRY sequence revealed that each of these markers were present in the library; the average number of positive clones per marker was 6.74 (range 2 to 12), consistent with 6.46 coverage of the tiger genome. Additionally, we identified 72 microsatellite markers that could potentially be used as genetic markers. This BAC library will serve as a valuable resource for physical mapping, comparative genomic study and large-scale genome sequencing in the tiger.

## 1. Introduction

Bacterial artificial chromosome (BAC) libraries are indispensable for physical analysis of large chromosomal regions, map-based gene isolation, and gene structure and function analysis [1,2]. It provides easy access to stable material for DNA manipulation such as exon trapping, cDNA selection, direct sequencing, microsatellite marker isolation, fluorescence in situ hybridization (FISH), and physical mapping [3,4]. BAC libraries have been constructed for several species of agricultural importance and wild animals, including cattle [5], bovine [6], goat [7], horse [8], pig [9,10], sheep [11], giant panda [12] and nurse shark [1].

Tiger (*Panthera tigris* Linnaeus, 1758) is the largest felid species and a widely recognized symbol of wildlife conservation. Historically tigers inhabited much of Asia, including the regions between the Caspian and Aral Seas, southeastern Russia, and the Sunda islands [13]. There are four generally accepted tiger subspecies in China: Siberian tigers (*P. t. altaica*), Indochinese tigers (*P. t. corbetti*), South China tigers (*P. t. amoyensis*), and Indian or Bengal tigers (*P. t. tigris*). It is estimated that fewer than 20 Siberian tigers, also known as the Amur tiger, exist in the wild in northeastern China [14]. The tiger is warranted the highest level of protection by the Convention on International Trade in Endangered Species of Wild Fauna & Flora (CITES). In 1989, the Chinese government placed the Siberian tiger into the highest National Protected Animal category.

However, up to now, only few functional genes and mitochondrial sequences of *Panthera tigris* have been cloned and partially studied according to the latest data in NCBI. And, to our knowledge, no BAC libraries exist for *P. t. altaica*. In previous studies, we cryopreserved Siberian tiger and Bengal tiger genomic resources by establishing fibroblasts cell lines. Moreover, study of normalized full-length cDNAs libraries and preliminary analysis of ESTs from Siberian tiger and Bengal tiger conducted in our laboratory were described previously [14,15].

Here, we describe the construction and characterization of Siberian tiger BAC library containing two sub-libraries prepared from blood cells and two sub-libraries prepared from fibroblasts. Additionally, 19 microsatellite markers and the SRY gene were used to screen the BAC library confirm its quality. We expect this key resource will facilitate further genetic studies in this important wild species.

## 2. Results

### 2.1. Cell Cultures and Characteristic Tests

**Figure 1.**
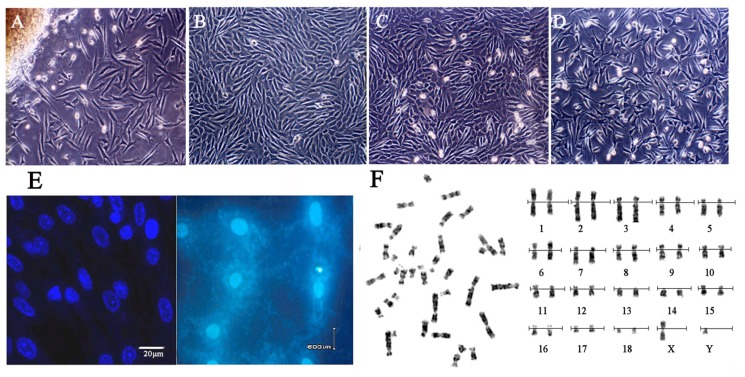
Morphology, Mycoplasma contamination and karyotype of Siberian tiger cell line. (**A**) Primary cells (100×), the cells were typical long spindle-shape; (**B**) Subcultured cells (100×); (**C**) Cells before cryopreservation (100×); (**D**) Cells after recovery (100×); (**E**) Mycoplasma detection for fibroblasts stained with Hoechst33258 and positive control of Mycoplasma contamination; (**F**) G-band chromosome at metaphase (**Left**) and karyotype (**Right**) (♂, 1000×).

We used a primary explanting technique and cell cryogenic preservation technology to establish the Siberian tiger fibroblast cell line and proceeded to biological and genetic detection. The culture conditions were optimal, and the cells were healthy (Figure 1A–D). Because we wanted to construct the BAC library to conserve the genome of the Siberian tiger, the fibroblasts must maintain diploid character similar with the cells *in vivo*. Chromosome analysis showed that the frequency of fibroblast cell chromosome number of 2*n* = 38 was 90.2%–91.6% in passage 1 to 3, which indicated that the cell line was still primarily diploid (Figure 1F). The test results of the bacteria, virus and mycoplasma screens were negative (Figure 1E).

### 2.2. Vector Preparation and High-Molecular-Weight (HMW) Genomic DNA Preparation

**Figure 2.**
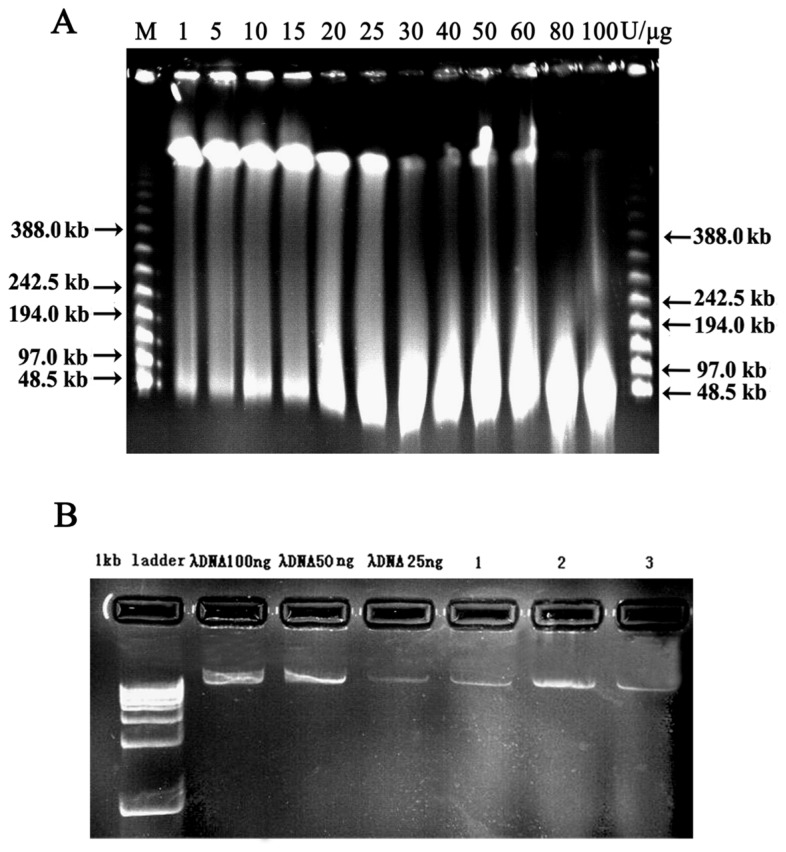
Characterization of digested BAC vector and PFGE size fractionation of digested HMW genomic DNA by *Hin*d III. (**A**) Initial PFGE size fractionation of partially digested HMW; M: Lambda Ladder PFG Marker; 1, 5, 10, 15, 20, 25, 30, 40, 50, 60, 80 and 100 U/μg DNA respectively; (**B**) Quantification of Siberian tiger genomic DNA. 1, 100–200 kb DNA; 2, 200–300 kb DNA; 3, 300–400 kb DNA.

The suitability of a BAC library for positional cloning depends on its genome coverage, average insert size, low percentage of mitochondrial DNA contamination, and low frequency of empty clones. An important step in constructing a library is isolation and partial digestion of high-molecular-weight DNA. Partial restriction enzymes digests were therefore optimized and assessed by monitoring the appearance of DNA smaller than 100 kb and the decrease in DNA in the high-molecular-weight (>1 Mbp) condensed zone (Figure 2A). We selected the insert DNA solution with the greatest concentration of high molecular weight DNA for use in subsequent ligation reactions. In our experience, 100–200, 200–300, 300–400 kb solutions that have DNA concentrations >5.0 ng/μL can be used in ligation (Figure 2B). If the insert solutions are particularly dilute (<5.0 ng/μL), they can be concentrated using Millipore nitrocellulose filters (Millipore Corporation, Billerica, MA, USA) and 10% (*v*/*v*) PEG8000. High MW (insert) DNA samples are quite unstable, so it is best to perform ligation immediately after checking the DNA concentration.

### 2.3. Characterization and Insert Size Testing of BAC Library

**Figure 3.**
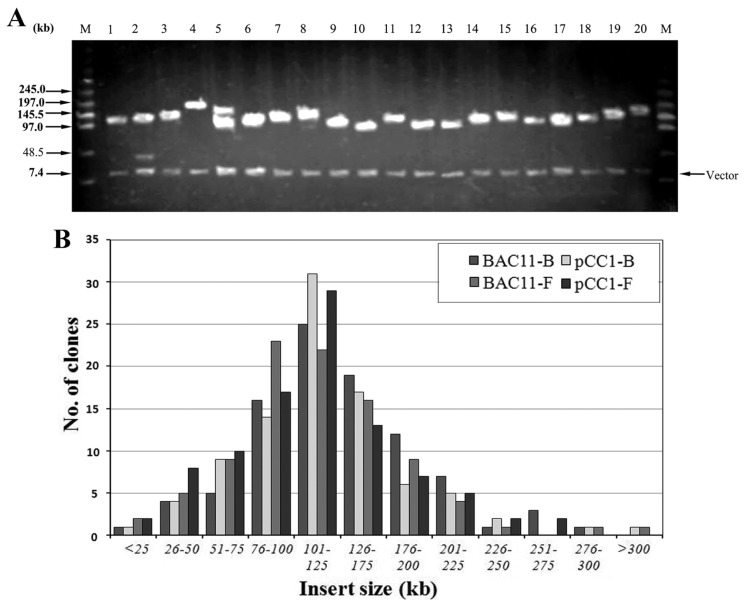
Characterization and Insert size testing of Siberian tiger BAC Library. (**A**) Analysis of the size of BAC clones by PFG electrophoresis. M: Ladder PFG Marker; Lanes 1–20: randomly picked recombinant BAC DNA digested with *Not*I; (**B**) BAC insert size distribution in the library. Insert sizes were determined from 384 BAC clones. The horizontal axis shows the size range in kb while the vertical axis displays the number of clone corresponding to each size range. Insert sizes are reported in a cumulative histogram.

The library contains approximately 153,600 clones, arrayed into 400 microtiter plates (384-well) using an automatic colony picker. Among these 153,600 clones, 115,200 were prepared from blood cells and 38,400 from fibroblast cells (Table 1). DNAs were prepared from 384 randomly selected clones, cleaved with *Not*I, and examined by PFGE to evaluate the size of the Siberian tiger genomic insert (Figure 3A).

Since there were no significant differences in the average insertion fragment lengths of the genomic BAC library from the four sub-libraries, we analyzed insert size of the BAC clones together. The average insert size of BAC clones was estimated to be 116.5 kb, with the small inserts (<75 kb) accounting for less than 15.8%, non-recombinants only 2.6%, 74.3% of the inserts ranging from 75 to 200 kb, and 65.1% greater than 100 kb (Figure 3B). Thus this library is approximately 6.46-fold genome equivalents with unbiased chromosomal distribution, representing a 98.86% statistical probability of obtaining at least one clone containing a unique DNA sequence in the library.

### 2.4. The Testing of Library Stability

**Figure 4.**
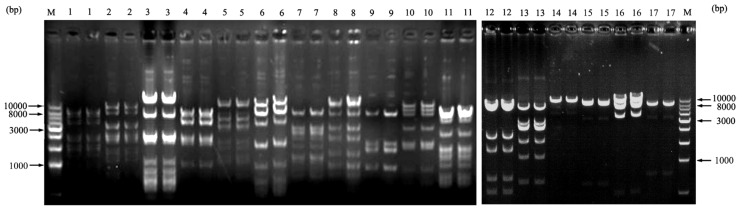
Examination of the BAC colony stability. M was 1 kb DNA Ladder; lanes 1–11 were restrictive finger print of different BAC clones from blood cell and 12–17 from fibroblast cells in second electrophoresis; Every clones has two electrophoretic bands, the first was at day 1 and the second was at day 5.

To assess library stability, 17 random BAC clones were assayed by serial culture for more than 100 generations over a period of 5 days. Every 24 h period was considered to represent about 20 generations [16]. The electrophoretic patterns of each clone digested with *Hin*d III were identical from days 1~5, confirming that the Siberian tiger BAC-clone inserts were stable after long-term culture (Figure 4).

### 2.5. BAC End Sequence Analysis

We sequenced the 480 sampling clones at both ends. 916 BAC end sequences (BES) were obtained with an average high quality base-pair number of 586 bp [17], covering a total of ~540,000 bp. A search for simple sequence repeats (SSRs) in the Siberian tiger BES dataset revealed 72 repeat sequences found in 69 BESs. The SSRs were deposited in GenBank under accession KF463188 to KF463259. The most frequent SSRs were dimers (25.0%) and trimers (59.7%), followed by tetramer monomer repeats (8.3%). Pentamer and hexamer repeats were present at much lower frequencies, accounting for only 7.0% of the microsatellites present. The microsatellite occurrence rate in the Siberian tiger genome seems to be approximately one SSR every 7.5 kb.

### 2.6. BAC Library Screening

To estimate the genome coverage of the BAC library by determining the number of clones that contain selected DNA markers or known functional genes [9], the BAC library was screened by PCR for 19 microsatellite markers and the *SRY* gene. Positive BACs for these markers and genes ranged from 2 to 12 with an average of 6.74 clones (Table 2). The average of 6.74 positive clones is compatible with the estimate of a 6.46-fold redundant library, and confirmed the library is unbiased. However, using our PCR-based screening protocol, the *SRY* gene was not found in the library.

## 3. Discussion

In the last 15 years, BAC libraries have been extensively used in physical mapping and complete eukaryote genome sequencing [10]. Previous studies have shown that a clonal coverage of 6.0–8.0 genome equivalents is sufficient for the development of a genome-wide physical map with approximately 95% genome coverage. We have successfully constructed a giant panda BAC library, which is large-insert, deep-coverage and publicly available. A Siberian tiger BAC library was constructed using blood cells and fibroblast cells from a male animal. The average insert size of Siberian tiger BAC clones was estimated to be 116.5 kb, representing approximately 6.46 genome equivalents.

BAC end sequences (BESs) and marker not only provide a snapshot of the sequence composition of the genome of the species of interest but also aid in genome assembly [3,18], chromosome walking [19], identifying genetic markers [20], and mapping genes to specific loci using fluorescence *in situ* hybridization (FISH) technology [21]. In this study, 916 BESs were obtained with an average high quality base-pair number of 586 bp, and 72 new SSRs were found in 66 BESs. We identified four microsatellite markers from the domestic cat genome (FCA201, FCA069, FCA043 and FCA094) in our tiger library. These new SSRs will be useful for Siberian tiger conservation genetic studies, for studies of genetic diversity, germplasm characterization and selection, and development of saturated genetic linkage maps.

The Siberian tiger BAC library was constructed from male DNA; thus, both X and Y chromosomes are represented, but each sex chromosome is underrepresented because only one copy of each is present in the genome with 2 copies of every autosome. This characteristic must be considered when using the library to screen genes on the X and the Y chromosomes that are not part of the pseudo-autosomal region.

Efficient library screening is crucial for all applications of the library. Screening can be performed either by hybridization on high-density filters or by the polymerase chain reaction (PCR). The main advantages of hybridization are the ability to combine probes for screening the entire BAC library and identifying clones in a single experiment [16]. PCR screening, however, is much more reliable, faster, and efficient with higher specificity owing to effective avoidance of false positive clones. Here, we used a four-step PCR screening procedure based on the BAC library pool system. BAC clones cultured overnight served as PCR template directly, rather than using prepared BAC-DNA. This modification considerably simplifies the procedure and shortens the time required for library screening. We identified 19 microsatellite markers that could potentially be used as genetic markers.

## 4. Materials and Methods

### 4.1. Preparation of BAC Vector

The BAC vector pBeloBAC11, provided by NEB company (New England Biolabs, Baverly, MA, USA), was isolated using the Qiagen Plasmid Mega Kit (Qiagen, Valencia, CA, USA) and purified by CsCl-ethidium bromide density gradient centrifugation, digested with the appropriate amount of *Hin*dIII (New England Biolabs, Baverly, MA, USA) and treated with alkaline phosphatase (CIAP) for dephosphorylation [22]. The vector was incubated with T4 DNA ligase, which circularized or joined vector molecules that had not been properly dephosphorylated. Electroelution was used to separate the linear dephosphorylated vector (7.5 kb) from non-dephosphorylated molecules.

### 4.2. High-Molecular-Weight (HMW) Genomic DNA Preparation

A fibroblast cell line generated from sixteen male Siberian tigers with normal karyotype was obtained using primary explanting techniques and cell cryogenic preservation technology [23]. The cell line was tested for viability, microorganism contamination, and chromosome euploidy according to Yasui *et al.* [24]. White blood cells and fibroblast cells from an adult male Siberian tiger were collected independently and mixed with 1% (*w*/*v*) Seaplaque GTG agarose (Cambrex, Rockland, ME, USA) at a concentration of 5 × 10^7^ cells/mL. The cell-agarose suspension was transferred into DNA plug molds to form solid agarose plugs.

The agarose plugs were treated with freshly prepared proteinase K digestion and then partially digested with *Hin*dIII restriction endonuclease (New England Biolabs, Baverly, MA, USA) as described in published protocols [25]. Using Lambda Ladder PFG Markers (New England Biolabs, Baverly, MA, USA) as a standard, the digested DNA plugs were subject to PFGE and the gel block containing 100–400 kb restriction fragments were cut in 0.5 cm slices. A second PFGE was then performed to remove small DNA fragments coiled within the large DNA fragments in the gel slices. The HMW DNAs were purified through electroelution and dialysis, and quantified by agarose gel electrophoresis with the λ DNA marker of known concentration.

### 4.3. Ligation and Transformation

Diluted DNA was quantitated and the ligated with dephosphorylated pBeloBAC11 vector or Copy Control pCC1 BAC (Epicentre, Madison, WI, USA) in a molar ratio of 5–10:1 in a 50 μL reaction volume containing 2 U T4 DNA ligase (Invitrogen, Carlsbad, CA, USA) at 16 °C overnight. The ligation mixture was dialyzed on a microdialysis filter (0.025 mm pore size; Millipore, Billerica, MA, USA). After ligation and microdialysation, 2 μL of the dialysed ligation product was used to electro-transform 20 μL of Electro MAX DH10B (Invitrogen, Carlsbad, CA, USA) or TransforMax EPI300 Electrocompetent *E. coli* cells (Epicentre, Madison, WI, USA) via a Gene Pulser apparatus (BTX-ECM630) at different voltages (~1.3–2.5 kV/cm) to maximize the transformation efficiency. After electroporation, transformants were incubated at 37 °C with continuous shaking for 1 h to allow recovery from electric shocks and expression of the antibiotic resistance gene within the transformed vectors. The cells were then plated on Luria–Bertani (LB) agar plates (100 mm × 15 mm) containing 20 μg chloramphenicol/mL and incubated at 37 °C for 16 h.

### 4.4. Large-Scale BAC Clone Production

The ligation was scaled-up under optimized conditions, and the transformed cells were pooled together for large-scale production. An automatic colony picker (QPIX II, Genetix, Hampshire, UK) was used to inoculate the BAC clones into 384-well microtiter plates with freezing media. The plates were incubated at 37 °C for 14–16 h to ensure the proper growth viability of the clones. The freezing media (36 mM K_2_HPO_4_, 13.2 mM KH_2_PO_4_, 1.7 mM sodium citrate, 0.4 mM MgSO_4_, 6.8 mM (NH4)_2_SO_4_, 4.4% *v*/*v* glycerol, and 12.5 μg/mL chloramphenicol) was used in the 384-well plates to protect the *E. coli* cells from damage under frozen conditions. After incubation and replication, the plates were stored in −80 °C freezers.

### 4.5. Insert Size Distribution of Siberian Tiger BAC Library

A total of 384 BAC clones (96 clones from each sub-library) were randomly picked from the Siberian tiger library and incubated in LB medium overnight at 37 °C. The BAC DNAs were isolated using a rapid alkaline lysis miniprep method, and digested individually at 37 °C for 3 h with 0.5 U *Not*I. The molecular weights of the BAC inserts were calculated according to the Lambda Ladder PFG Marker (New England Biolabs, Baverly, MA, USA).

### 4.6. Library Pooling and BAC Library Screening

To establish the two-step PCR screening systems, the library was divided into 20 superpools where one superpool was comprised of twenty 384-well plates. Cultures from every superpool were combined to make superpool DNA for the first step PCR screening. In each superpool, cultures from each plate (384 clones), row (24 clones × 20 plates) and column (16 clones × 20 plates) were combined respectively to make DNA for the second step screening.

To assess the quality of this library further, 19 microsatellite markers selected from different regions of tiger genome and the SRY gene were used for screening. The 19 microsatellite markers, including four microsatellite loci-FCA201, FCA069, FCA043 and FCA094 from the cat genome, were identified in the tiger genome. BAC screening was performed by two-step PCR (superpools PCR and 4D-PCR) [12]. Positive BAC clones were confirmed by the fragments amplified for the expected size and sequencing of PCR products. After trimming off the vector sequences, the sequences of insert DNAs were BLASTed against the cat genome sequences (http://www.ncbi.nlm.nih.gov/blast).

### 4.7. BAC End Sequencing

Four hundred and eighty BAC sampling clones (192 clones from BAC11-B, 96 clones extracted from pCC1-B, BAC11-F and pCC1-F, respectively) were sequenced at both ends using BigDye Terminator v.3 (Applied Biosystems, ABI, Foster City, CA, USA) according to manufacturer’s instruction. The T7 primer (5′-TAATACGACTCACTATAGGG-3′) and BES_HR primer (5′-CACTCATTAGGCACCCC A-3′) were used as forward and reverse primers. BAC clone end sequencing (BES) using the method of Luo *et al.* [1], and the resultant sequences were trimmed vector and low quality sequences and analyzed for mitochondrial genome contaminations using BLASTN searches.

## 5. Conclusions

The first high-quality, representative Siberian tiger genomic BAC library has been constructed, covering about 6.46-fold genome equivalents of the tiger genome. There were no significant differences of genomic BAC library from blood cells and fibroblast cells. These 72 new SSRs will be very useful for Siberian tiger conservation genetic studies. The availability of the Siberian tiger BAC library will aid in identification of genes and genomic regions of interest, development of genetic markers, and genome characterization via BAC end sequencing and deep sequencing of selected clones.

## Figures and Tables

**Table 1.** Summary of the Siberian tiger BAC library.

Sub-library	Sample	Vector	Bacterial strain	Cloning enzyme	Total clones	Plate No.	Non-insert clone (%)	Average insert size a (kb)
BAC11-B	Blood cells	pBeloBAC11	*E. coli* DH10B	*Hin*dIII	92,160	1–240	2.1 (2)	126.5
pCC1-B	Blood cells	Copy Control™ pCC1 BAC™	EPI300™-T1^R^ *E. coli*	*Hin*dIII	23,040	241–300	5.2 (5)	116.7
BAC11-F	Fibroblasts	pBeloBAC11	*E. coli* DH10B	*Hin*dIII	23,040	301–360	3.1 (3)	114.3
pCC1-F	Fibroblasts	Copy Control™ pCC1 BAC™	EPI300™-T1^R^ *E. coli*	*Hin*dIII	15,360	361–400	1.0 (1)	110.1
Total					153,600	400	2.6	116.5

Genome coverage 6.46×; Probability of finding a target gene was 98.86%;

a Determined using random clones from ligations.

**Table 2.** Summary of results from screening the Siberian tiger BAC library by PCR with primers for microsatellites. The number of positive superpools for each locus (NF: not found) and the number of positive BAC clones are listed (Blood/Fibroblasts).

Marker name	Location Chr	Number of positive superpools	Number of positive clones
KF463190	A1	4/2	6/2
KF463196	A2	3/1	4/1
KF463200	A3	3/0	9/3
KF463203	B1	2/0	5/2
KF463210	B2	1/2	4/2
FCA201	B3	3/2	4/3
FCA069	B4	3/1	4/1
KF463223	C1	3/NF	6/0
FCA043	C2	3/1	5/2
KF463232	D1	4/1	6/1
KF463233	D2	2/2	4/4
KF463238	D3	2/3	5/3
KF463238	D4	NF/2	0/2
KF463245	E1	4/1	8/1
KF463247	E2	3/1	5/2
KF463252	E3	3/2	5/2
KF463255	F1	2/NF	4/1
FCA094	F2	4/2	5/1
KF463257	X	2/1	3/1
SRY	Y	1/0	1/0
Average number of positive clone			4.90/1.84
